# Long term results and quality of life after primary excision followed by Limberg plasty in pilonidal sinus disease

**DOI:** 10.1007/s00423-025-03776-8

**Published:** 2025-06-14

**Authors:** Jenny König, Utz Settmacher, Aladdin Ali Deeb, Oliver Rohland, Felix Dondorf, Astrid Bauschke, Falk Rauchfuß, Michael Ardelt

**Affiliations:** 1 Friedrich-Schiller University, Jena, Germany; 2 Department of General, Visceral and Vascular Surgery, Jena University Hospital, Jena, Germany

**Keywords:** Limberg plasty, Pilonidal sinus disease

## Abstract

**Background:**

Pilonidal sinus disease (PSD) is a frequently occurring condition that can have a significant impact on quality of life (QoL). In addition to severe pain, particularly with movement or while sitting, this disease imposes restrictions that affect one’s professional and private life. These patients frequently suffer from recurrence requiring multiple interventions and hospital stays.

**Methods:**

The study enrolled forty-seven patients who presented with PSD from August 2010 until June 2019 and underwent primary excision and LP at the Department of General, Visceral, and Vascular Surgery at University Hospital, Jena. Forty- one of these patients were questioned retrospectively in writing or by telephone interviews between July 2021 to September 2022. The data processed using SPSS software.

**Results:**

The median follow-up for all patients was 86 months (range, 23–140 months). Only one recurrence (2.4%) was reported. While the participants’ BMIs remained unchanged, they reported significant improvements in QoL, notably in five of the six activities of daily living that were evaluated.

**Conclusion:**

The low rate of recurrence suggests that LP is an effective option for post-excision surgical repair of pilonidal sinus. The use of this procedure has no impact on patient’s BMI but can significantly improve patients’ QoL.

## Background

Pilonidal sinus disease (PSD) is an acquired condition involving an acute abscess and chronic inflammation in the sacrococcygeal area [1]. There are numerous treatment options available to treat this painful and unsightly condition. Because of high rates of recurrence and complications that frequently results when this condition is managed with conservative methods, including phenol instillation [2] or local radiation, these strategies are no longer recommended [3]. Instead, surgical techniques can be used to address this condition, including pit picking [4], endoscopic procedures, and singular excision, as well as two-stage methods that include excision followed by cosmetic surgical repair after an inflammation-free interval [5]. Based on the then applicable AWMF guidelines from 2008 to 2014, the patients underwent excision and plastic coverage in an infection-free interval [6, 7].

Karydakis (KP) [8] or LP [9] procedures are advocated for flap creation and transposition [7, 10].

**Fig. 1:**
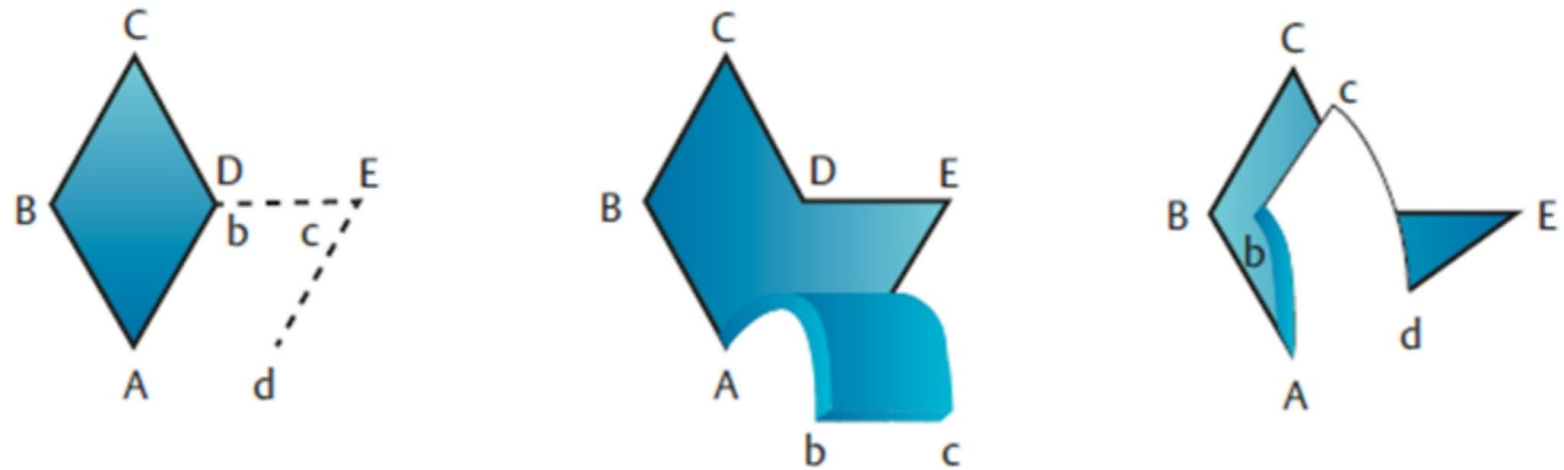
Schematic image of the Limberg flap. Following the mobilization of the flap, it is being moved around the angle point **A** into the rhombic shaped defect. The corners that are designated with lower case letters are being placed at the respective corners of the defect that are designated with capital letters (b on **B**, c on **C** and d on **D**) [10].

In the two-phase concept, radical excision is performed first. The entire fistula system is marked using methylene blue and then completely excised so that the resection margins do not show any blue marking. Plastic defect coverage is then performed in the inflammation-free interval. The LP procedure involves suture lateralization with simultaneous flattening of the rima ani to promote healing and prevent recurrence (surgical technique see Fig. 1) [11, 12]. In the most frequently performed of the modified versions of this technique, both the superior and inferior edges of the wound pole are positioned laterally to the rima ani [13]. This modification addresses problems associated with the original flap transposition procedure. The earlier procedure led to recurrence rates as high as 20% because the inferior wound pole crossed over the rima ani [14]. In addition to reducing the rate of disease recurrence, prevention of pilonidal sinus carcinoma is also a concern. Recurrent or chronic inflammation is associated with an increased risk of developing malignancies. Long-term inflammatory processes can lead to changes in the tissue that promote tumor growth. The prognosis of coccyx fistula carcinomas is also worse than that of squamous cell carcinomas [15]. In 2023, Esposito et al. reported that malignancy develops in only 0.1% of patients with PSD. However, those patients who do develop pilonidal malignancy do not respond well to radiation or chemotherapy. Surgical intervention must be considered not only to prevent recurrences but also to reduce the likelihood of further complications [16].

## Methods

### Data analysis

Participants in this study included patients who underwent surgery for International Classification of Diseases (ICD) L.05-L.05.9 (pilonidal cysts with or without abscess) between August 2010 and June 2019 at the Department of General, Visceral and Vascular Surgery at the University Hospital Jena. Because of the restrictions imposed by COVID-19 pandemic, retrospective data were collected by telephone interview, email, and regular mail between July 2021 and September 2022. The participants’ body weights and heights were retrieved from SAP (“Systemanalyse Programmentwicklung”) for the calculation of body mass indices (BMIs). These parameters were also recorded pre-operatively in the premedication outpatient clinic or on the patient ward.

**Fig. 2:**
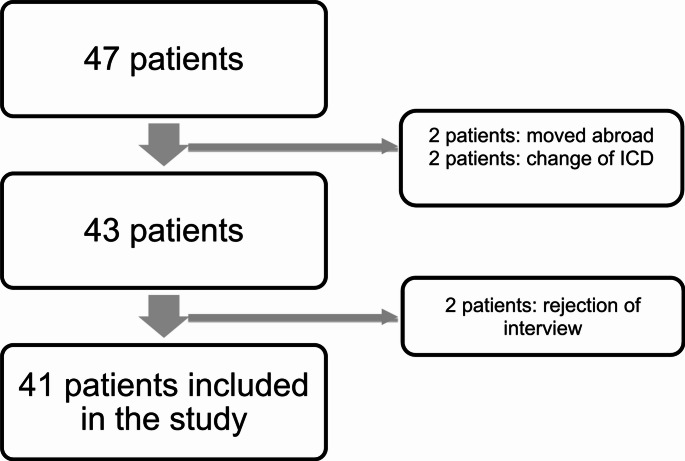
Flow chart of patient recruitment

Six of the original 47 patients identified as eligible for the study were excluded from the data collection process. Of these six, two patients declined to participate, two had moved abroad, and two experienced complications that required changes to their ICD codes (osteomyelitis and suture granuloma, see Fig. 2). In the two patients with ICD changes, the LP was dissolved and excision was performed. We determined the recurrence rate and compared our findings to those reported in the literature. We also evaluated the impact of LP on postoperative BMIs and quality of life (QoL). For the latter assessment, the Moorhead-Ardelt Quality of Life Questionnaire (MAQoLQII) was used retrospectively. The study was approved by the ethic committee (ethics application number: 4653-01/16).

## Quantitative evaluation

We performed quantitative analysis on data collected from the 41 study participants. We performed calculations and determined statistical significance using SPSS software (IBM SPSS Statistics 27). Pre- and postoperative median BMIs were compared using the Wilcoxon test; *P* <.05 was considered statistically significant. Patients’ responses to questions one to six of the MAQoLQII (individually and in total) regarding their pre- and postoperative states were evaluated respectively, with *P* <.05 considered statistically significant.

## Results

Forty-seven patients who presented with sacrococcygeal PSD underwent the two-stage procedure with primary excision followed by a secondary LP procedure during an inflammation-free interval. Following excision and discharge of the patient from hospital, wound management is being continued at home. Patients are being instructed to rinse the wound several times a day (5–7 times) with tap water. In addition, appointments for weekly wound control at the hospital are being made. As soon as clean wound conditions without infections are visible, and granulation is underway, plastic surgery for coverage with LP is being performed. Forty-one patients (40 male, and one female) were included in the study (Table 1). The median follow-up period was 86 months (range, 23–140 months).

**Table 1:** Demographic details of the study sample (*n* = 41 patients), interquartile area and percentages in brackets

Variable	Distribution (*n* = 41)
Age in years	25,0 (21,5; 29,0)
Male sex	40 (97,6%)
Smoker	25 (61%)
Chronic	11 (26,9%)
At least one recurrence prior to LP	26 (63,4%)
Recurrence after LP	1 (2,4%)
Follow-up in months	86 (46,5; 122)
Time period between surgeries in days	42 (28,5; 49,5)
Hospitalisation in days	4 (3; 4,5)

The disease was acutely abscess-forming in 30 patients (73.2%). The remaining 11 patients (26.8%) presented with signs of chronic disease. The median patient age at the time of the LP procedure was 25 years (range, 17–48 years). The median preoperative BMI was 27.78 kg/m^2^ (range, 18.94–40.04 kg/m^2^). Twenty-five patients (61%) were smokers. The median number of pre-procedure recurrences in this patient cohort was one (range, 0–10).

The median time elapsed between the primary excision and LP procedure was 42 days (range, 20–67 days) and the median postoperative hospital stay following LP was four days (range, 2–10 days). One patient experienced a recurrence during the follow-up period. According to Sondenaa et al., this event is defined in the S3 guideline as “Recurrence of pilonidal sinus with typical symptoms, unrelated to the initial wound healing” [10].

**Fig. 3:**
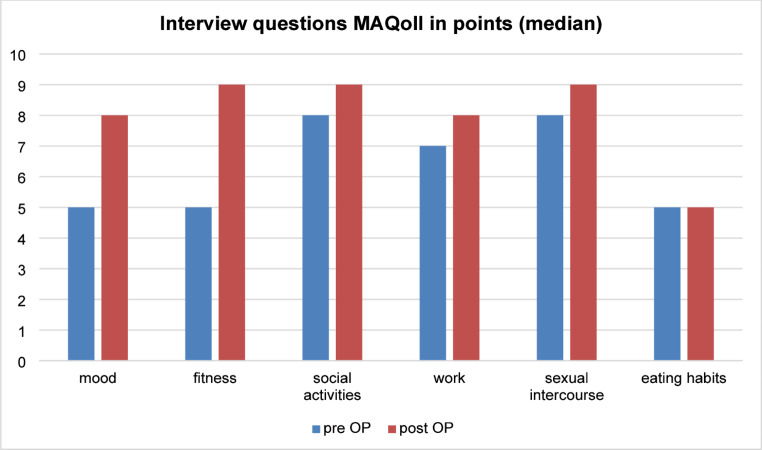
Median score of interview questions one to six of the Moorehead-Ardelt Quality of Life Questionnaire (MAQoLQII) in points pre- and postoperatively

The findings shown in Table 2 include median values and the statistical significance of differences in BMI determined pre- and postoperatively (the latter by questionnaire). Our results indicate that the surgical procedure had no significant impact on patients’ BMI over this time (*P* =.57). By contrast, our findings revealed a significant increase in total QoL score (*P* <.001) as well as in several of the individual areas addressed by this questionnaire, including mood, fitness, social life, professional work, and sexual intercourse (*P* <.001). There was no evidence of significant changes in nutrition intake (*P* =.57). Of note, the QoL assessment was performed using information from only 39 of the 41 patients enrolled in the study; two patients declined to provide this information. Figure 3 documents the median pre- and postoperative MAQoLQII values recorded for the individual areas of life.

**Table 2:** Outcomes of median BMI and point score of individual questions and sum of Moorehead-Ardelt quality of life questionnaire (MAQoLQII) pre- and postoperatively, as well as difference and significance of BMI-alterations (Wilcoxon test), an outcome *P* <.05 was considered significant

Variable	Pre OP points	Post OP points	Difference post-pre	Significance
BMI	27,8 (24,9; 34,1)	27,8 (24,8; 33,0)	0	*P* =.569
Question 1 - mood	5 (3;6)	8 (7;9)	3 (2;5)	*P* <.001
Question 2 - fitness	5 (3;6)	9 (8;10)	4 (2;5)	*P* <.001
Question 3 – social activities	8 (6;9)	9 (8;10)	1 (0;2)	*P* <.001
Question 4 - work	7 (7;8)	8 (7;9)	1 (0;2)	*P* <.001
Question 5 - sexual intercourse	8 (6;9)	9 (8;10)	1 (0;3)	*P* <.001
Question 6 – eating habits	5 (4;7)	5 (4;7)	0 (−1;0)	*P* =.571
Sum of questions	38 (33;41)	48 (45;51)	10 (6;13)	*P* <.001

## Discussion

According to Bi, Sun et al., the LP is the procedure used most frequently for post-excision cosmetic repair of PSD [17]. Several different post-LP recurrence rates have been reported in the literature, ranging from 0% (in 74 months follow-up up) to 20% (after 20 months) [14, 18]. Of note, the surgical technique used in each case is of crucial importance as the recurrence rates associated with the modified method [19] are markedly lower than those reported for the original LP technique. This is also why the German pilonidal sinus AWMF-S3-guideline states that the use of a midline closure for primary wound coverage should be avoided under all circumstances [19]. Furthermore, long follow-up periods are useful, because recurrences have been reported as long as 20 years after the initial procedure. Doll et al. reported a 17% recurrence rate at a median time of 1.8 years [20]. The single disease recurrence reported in our study occurred 59 months after the LP procedure. The recurrence rate after a KP was reported to be between 0% [21] and 13% (at 49 and 37 months follow-up, respectively) [22]. Minor complications developed in as many as 20% of patients, including seroma formation. By contrast, only 5% of patients exhibited these complications after an LP procedure [23]. However, at the current time, neither procedure has been identified as providing overall advantages [19]. Although the Karydakis procedure has been associated with a higher rate of minor complications (e.g., seromas and wound infections), patients who have undergone this procedure required shorter absences from work and reported higher satisfaction with the cosmetic results [24, 25]. Nevertheless, LP also leads to good patient satisfaction with acceptable cosmetic results [26]. The choice of procedure depends on various factors, including the skills of the surgeon. According to German AWMF S3 guidelines, both procedures have a recommendation grade of A [19].

In contrast to previous versions, the current German guideline does not recommend excision, except in exceptional cases in the acute abscessed stage. In the recent literature, treatment by incision or aspiration shows good results in 95% of PSD cases [10, 27, 28].

For this reason, this less invasive treatment with two-stage treatment is recommended in the latest german guideline [10]. 

PSD has been reported as developing 2.2 times more frequently in males than in females [29]. In our cohort, 98% of patients were men. Among the reasons for the skew, female patients may be reluctant to undergo extended plastic surgical procedures [5].

While our findings revealed no significant changes in pre- versus postoperative BMIs overall, patients who presented as overweight or obese preoperatively experienced greater weight loss over time. By contrast, patient’s QoL was severely impaired preoperatively due to PSD. The MAQoLQII results revealed that patients who underwent LP were markedly more mobile, in particular with respect to physical activity. Thus, one might conclude that weight loss could be achieved by an increase in physical exercise. Ertan et al. were also able to confirm good patient comfort and satisfaction with LP [30]. Others showed an average general life satisfaction of 6.7 (on a scale of 0–10) [31]. However, our patients reported improvements in all areas of everyday living, except their eating habits. Changes in this QoL area were not expected, as the disease itself has no direct impact on food intake.

The questionnaire used in this study was originally developed for use by bariatric specialties. This tool was also feasible for use in this evaluation as it is simple, easy to understand, and user-friendly, and can be completed in less than one minute [32, 33]. Furthermore, most of the patients enrolled in our study (75.6%) were initially overweight. We focused on the time required to complete the questionnaire, as this plays a decisive role in the reasonableness of our request and the use of patients’ resources [34]. Other questionnaires that we considered were too extensive or inappropriate for the evaluation of patient QoL in this study. For example, although the Messick Eating Inventory includes 17 questions on eating habits and has an elaborate image, it does not solicit information on other areas of everyday life [35]. Likewise, the Short Form (SF)−36 is even more elaborate and requires more time to complete (approximately seven minutes as per Cella et al.) [36, 37]. Furthermore, the Crohnbach’s alpha of the SF-36 is markedly below that calculated for the MAQoLII (0.817–0.885); the internal consistency of the questionnaire used in our study was also declared to be good [38, 39]. In 2005, a study was published that compared QoL after primary wound closure with that experienced after a rhombic flap procedure. The participants included 100 patients who were questioned using the SF-36 and other tools. Patient satisfaction with the LP procedure was confirmed in this study and an improved QoL was achieved [30].

## Limitations

This study has several limitations. The operations were performed over almost 10 years by the same surgeon and according to a standardized protocol. On the one hand, this ensures a high level of consistency, but on the other hand, it can lead to bias as surgical experience changes. The time interval between the LP procedure and the administration of the questionnaire was too long in some cases. Patients’ memories may be inadequate or impaired which may result in distortions when completing the MAQoLII. Similarly, the study cohort is somewhat small. This factor relates directly to the number of procedures performed and the fact that patients could only be recruited if they met the systemic definition of the disease and German guidelines. Two patients declined to participate in the study, thus reducing the cohort of patients. Most of the patients presenting with chronic PSD undergo elective surgery because of the limited likelihood of spontaneous healing. According to the German S3 guideline, this is a standard indication for open excision [10]. In 2019, Peters reported that this remained the most frequently applied procedure [40]. Due to the restrictions in times of the Covid-19 pandemic, postoperative data could only be collected by e-mail or telephone survey. Direct, personal contact was not permitted due to legal restrictions. The availability of surgeons who are capable of performing LP procedures remains uncertain. The results of a Danish study revealed that elective procedures for PSD were performed by just one or two surgeons in 11 of 37 hospitals (39%) [41]. Similarly, Peters’ 2019 study reported that while interns in Germany typically perform 75% of the straightforward open procedures, only 6% perform the follow-up plastic surgical repair [40]. Furthermore, only 15% of the attending and specialist surgeons reported that they performed this procedure. Finally, the fourth and fifth questions in the MAQoLII address rather private and sensitive personal areas. This may provoke incorrect answers to the questions to “(avoid embarrassment […])” [42]. Still, results from the prospective study published by Still, Pronk et al. reported significant improvements in preoperative sexual dysfunction at 6 and 12 weeks postoperatively [43]. A further limitation is that the guideline regarding excision in the acutely abscessed stage changed during the period of the study and is no longer recommended, except in exceptional cases [10].

## Conclusion

The two-stage procedure that included primary excision followed by LP during the inflammation-free interval results in significant improvements in the long-term QoL of patients presenting with PSD. The low recurrence rate observed in this study confirms the recommendation of the German S3 guideline for plastic surgical repair of these lesions.

## Data Availability

No datasets were generated or analysed during the current study.
